# Thyroid dose‐volume thresholds for the risk of radiation‐related hypothyroidism in nasopharyngeal carcinoma treated with intensity‐modulated radiotherapy—A single‐institution study

**DOI:** 10.1002/cam4.2574

**Published:** 2019-09-27

**Authors:** Cheng‐Long Huang, Hong‐Wen Tan, Rui Guo, Yuan Zhang, Hao Peng, Liang Peng, Ai‐Hua Lin, Yan‐Ping Mao, Ying Sun, Jun Ma, Ling‐Long Tang

**Affiliations:** ^1^ Department of Radiation Oncology Sun Yat‐sen University Cancer Center State Key Laboratory of Oncology in South China Collaborative Innovation Center for Cancer Medicine Guangdong Key Laboratory of Nasopharyngeal Carcinoma Diagnosis and Therapy Guangzhou People's Republic of China; ^2^ Ji'an Central People's Hospital Ji'an People's Republic of China; ^3^ Department of Medical Statistics and Epidemiology School of Public Health Sun Yat‐sen University Guangzhou China

**Keywords:** hypothyroidism, intensity‐modulated radiotherapy, nasopharyngeal carcinoma, prevalence, risk factors

## Abstract

**Background:**

To identify thyroid dose‐volume thresholds for radiotherapy (RT)‐related hypothyroidism (HT) in patients with nasopharyngeal carcinoma (NPC) treated with intensity‐modulated RT (IMRT). In this way, we desired to guide the design of treatment plans and, finally, lower HT prevalence.

**Methods:**

In total, 345 NPC patients treated with IMRT were evaluated retrospectively during a median follow‐up of 45.2 (range, 11.3‐64.9) months. Serum‐based assessments of thyroid function before and after IMRT were monitored periodically. Thyroid dose‐volume parameters were analyzed for their association with HT risk.

**Results:**

In total, 44.1% of patients (152/345) developed primary HT. Analyses of thyroid dose‐volume parameters identified a stringent dose‐volume histogram (DVH) threshold defined by *V*_25Gy_ (the percentage thyroid volume that receives >25 Gy, not the absolute volume) ≤60%, *V*_35Gy_ ≤ 55%, and *V*_45Gy_ ≤ 45%. Patients whose thyroid DVHs satisfied these constraints had a lower prevalence of 2‐year HT compared with the overall prevalence (13.2% vs 25.8%, *P* < .001). Another DVH was defined by *V*_25Gy_ > 95%, *V*_35Gy_ > 90%, and *V*_45Gy_ > 75%, and patients whose thyroid DVHs satisfied with these constraints had a higher prevalence of 2‐year HT than the overall incidence (36.0% vs 25.8%, *P* < .001).

**Conclusion:**

We recommend *V*_25Gy_ ≤ 60%, *V*_35Gy_ ≤ 55%, and *V*_45Gy_ ≤ 45% as the “stringent” DVH line, and *V*_25Gy_ > 95%, *V*_35Gy_ > 90%, and *V*_45Gy_ > 75% as the “inhibition” DVH line, under the precondition of not compromising the target coverage. These findings could help in the design of individual treatment plans and, eventually, to lowering of HT prevalence.

## Graphical abstract

The risk of radiation‐related hypothyroidism (RHT) varied significantly depending on the dose‐volume parameters of thyroid radiation. We investigated and identified multiple thyroid dose‐volume thresholds for RHT in nasopharyngeal carcinomas treated with intensity‐modulated radiotherapy (RT), to guide risk stratification of RHT and help in the design of individual RT plans.

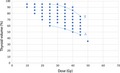

## 1 INTRODUCTION

Nasopharyngeal carcinoma (NPC) is especially endemic in Southern China, where the annual incidence is 30‐80 per 100 000 population.^1^ Radiotherapy (RT) is the primary treatment for NPC.^2^ The radiation target routinely includes the primary tumor, retropharyngeal area, and whole neck (levels II‐V),^3^ resulting in unavoidable irradiation to part of the thyroid gland. Scholars have reported that the prevalence of RT‐related hypothyroidism (HT) is 22‐29%.^4^, ^5^ Hypothyroidism may manifest as fatigue, cold intolerance, dry skin, weight gain, constipation, or no symptom that leads to different degrees of impact on function and quality of life.

Several studies have investigated the risk factors for HT after intensity‐modulated radiation therapy (IMRT) for nonmetastatic NPC.^4^, ^6^, ^7^ Zhai et al recommended to reduce thyroid V_45Gy_ (the percentage thyroid volume that receives >45 Gy, not the absolute volume) to 50% and *V*_50_ to 35%.^4^ Sommat et al reported that thyroid *V*_40Gy_ ≤ 85% was a useful dose constraint during IMRT plans without compromising tumor coverage.^7^ Lee et al revealed that VS_60Gy_ (the absolute thyroid volume spared from ≤60 Gy) ≥10 cm^3^ and VS_45Gy_ ≥ 5 cm^3^ could lower the risk of HT without compromising target coverage.^6^ These dose‐volume thresholds helped clinicians design more appropriate treatment plans. However, the studies mentioned above focused only on one or two thresholds that might not always be applicable to individuals. That is, once the tumor volume, especially the metastatic lymph node, is large and the target must be expanded, the thyroid gland will receive a high dose unavoidably, and such thresholds might not be applicable. Hence, more constraints are needed to guide the design of individual treatment plans.

In the present study, we investigated thyroid dose‐volume thresholds for the risk of HT in NPC treated with IMRT. In this way, we desired to identify multiple dose‐volume thresholds to guide risk stratification of RT‐related HT and, finally, lower the prevalence of HT.

## 2 MATERIALS AND METHODS

### 2.1 Patients

The study protocol was approved by the Ethics Committee of our hospital. NPC patients treated at our cancer center between 2012 and 2016 were reviewed retrospectively. The inclusion criteria were as follows: (a) newly diagnosed, previously untreated, and pathologically confirmed World Health Organization type‐II or ‐III NPC; (b) no distant metastasis; (c) Karnofsky Performance Scale score >60; (d) no abnormal thyroid function before RT; (e) radical IMRT was planned. Exclusion criteria were patients: (a) with previous thyroid surgery; (b) with preexisting pituitary disorders; (c) who had received irradiation to pituitary/parasellar tumors, or head and neck, or the whole body previously. Finally, 345 NPC patients (249 males and 96 females; median age, 44 years; range, 7‐81 years) were included retrospectively. The characteristics of the included patients are shown in Table 1. All included patients had been evaluated by complete physical examination, chest radiography, electrocardiography, abdominal sonography, emission computed tomography, magnetic resonance imaging (nasopharyngeal and neck), and blood samples for thyroid‐function assessment. The authenticity of this article has been validated by uploading the key raw data onto the Research Data Deposit public platform (https://www.researchdata.org.cn), with the approval RDD number as RDDA2019001173.

**Table 1:** Characteristics of included patients

Characteristic	Number (%)
Age (y)	
>44	163 (47.2)
≤44	182 (52.8)
Sex	
Female	96 (27.8)
Male	249 (72.2)
Pretreatment thyroid volume, cm^3^	
>16	178 (51.6)
≤16	167 (48.4)
WHO pathologic type	
Undifferentiated nonkeratinizing	343 (99.4)
Differentiated nonkeratinizing	2 (0.6)
Tumor classification^a^	
T1	59 (17.1)
T2	68 (19.7)
T3	147 (42.6)
T4	71 (20.6)
Node classification^a^	
N0	54 (15.7)
N1	167 (48.4)
N2	79 (22.9)
N3	45 (13.0)
Overall stage^a^	
I	23 (6.7)
II	67 (19.4)
III	150 (43.5)
IV	105 (30.4)
Mean radiation dose (Gy)	
>47	162 (47.0)
≤47	183 (53.0)
Maximum radiation dose (Gy)	
>63	155 (44.9)
≤63	190 (55.1)
Minimum radiation dose (Gy)	
>26	172 (49.9)
≤26	173 (50.1)
Chemotherapy	
Yes	293 (84.9)
No	52 (15.1)

Abbreviation: WHO, World Health Organization.

a According to the Union for International Cancer Control/American Joint Committee on Cancer criteria (8th version).

### 2.2 Treatment

All patients were treated with IMRT. Delineation of the target volume was in accordance with the treatment protocol of our institution^8^ and the International Commission on Radiation Units and Measurements reports 50 and 62. All targets were treated simultaneously using the simultaneous integrated boost technique. Intensity‐modulated RT was generated for an Elekta and Varian linear accelerator using 6 MV photons, and delivered in the step‐and‐shoot and sliding window mode. Dose optimization and calculation for IMRT plan were performed on the Monaco treatment planning system (version 3.02; Elekta Medical Systems) using the Monte Carlo algorithm and eclipse treatment planning system (version 11.0; Varian Medical Systems) using the AAA algorithm. The prescribed doses were 66‐72 Gy/28‐33 fractions to the planning target volume (PTV) of the primary gross tumor volume (GTVnx), 64‐70 Gy/28‐33 fractions to the GTV PTV of the involved lymph nodes (GTVnd), 60‐63 Gy/28‐33 fractions to the high‐risk clinical target volume PTV (CTV1), and 54‐56 Gy/28‐33 fractions to the low‐risk clinical target volume PTV (CTV2). Overall, 293 (84.9%) patients received platinum‐based neoadjuvant, concomitant, or adjuvant chemotherapy, whereas 52 (15.1%) patients did not.

### 2.3 DVH parameters of the thyroid gland

Dose‐volume histograms (DVHs) for the thyroid gland were computed from the three‐dimensional (3D) dose distributions and exported from treatment plans of the Monaco treatment planning system (version 3.02; Elekta Medical Systems) and eclipse treatment planning system (version 11.0; Varian Medical Systems). We investigate potential threshold doses in 5 Gy increments, ranged from 5 to 70 Gy, and the percentages of thyroid volume that received more than one of these potential threshold doses of radiation (VDose) were calculated.

### 2.4 Evaluation of HT

Thyroid function, including serum levels of thyroid‐stimulating hormone (TSH), free thyroxine (FT4), and free triiodothyronine (FT3), was evaluated before and after RT, every 3 months during the first year, every 6 months in the second to fifth year, and annually thereafter. The median duration of follow‐up was 45.2 (range, 11.3‐64.9) months. The electrochemiluminescence method employing an Elecsys 2010 analyzer (Roche Laboratory Systems) was used. At our institution, the reference range for TSH was 0.27‐4.20 µIU/mL, for FT4 was 12.00‐22.00 pmol/L, and for FT3 was 2.80‐7.10 pmol/L. “Primary HT” was defined as a serum concentration of TSH > 4.20 µIU/mL, with a normal or low level of FT4. “Clinical HT” was defined as an increased TSH level (>4.20 µIU/mL), with a low level of FT4.

### 2.5 Statistical analyses

The study endpoint was primary HT. Time to HT was calculated from the start of RT and was censored at final follow‐up if patients did not experience the endpoint. Statistical analyses were undertaken using SPSS v20.0 (IBM). *P* < .05 was considered significant.

We used the log‐rank test to carry out univariate analysis of differences in time to HT in subgroups of patients divided by factors such as age (>44 vs ≤44 years, divided according to the median age), sex, pretreatment thyroid volume (>16 vs ≤16 cm^3^, divided according to the median thyroid volume), T classification (T1‐2 vs T3‐4), N classification (N0 vs N1‐3), overall stage (stage I‐II vs stage III‐IV), thyroid mean radiation dose (>47 vs ≤47 Gy, divided according to the median value), maximum radiation dose (>63 vs ≤63 Gy, divided according to the median dose), minimum radiation dose (>26 vs ≤26 Gy, divided according to the median value), use of chemotherapy (yes vs no), and multiple dose‐volume parameters from thyroid DVH curves.

Specifically, to identify the threshold dose (Dose) and percent thyroid volume (%*V*), time to HT was compared in subgroups with *V*_Dose_ ≤ %*V* vs *V*_Dose_ > %*V*. Threshold doses ranged from 5 Gy (*V*_5Gy_) to 70 Gy (*V*_70Gy_) in increments of 5 Gy, and thyroid volumes (%*V*) ranged from 5% to 95% in increments of 5%. To ensure data robustness, analysis was only carried out if a subgroup contained more than 25 patients. Also, to limit the possibility of finding spurious significant associations because of the large number of analyses carried out, a Bonferroni correction was applied to nominal *P*‐values; comparisons were considered significant only if *P* was <.05/n, where n is the total number of dose‐volume parameters tested (n = 133). Multivariate analysis was undertaken using the Cox proportional hazards model.

## 3 RESULTS

### 3.1 Characteristics and univariate analysis

**Figure 1:**
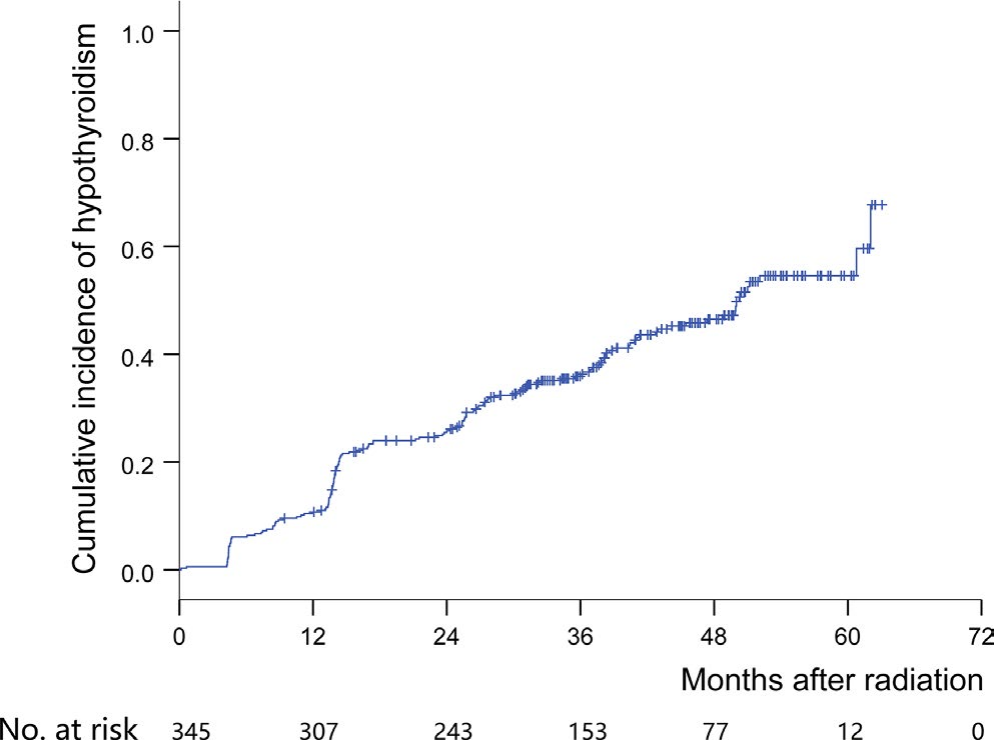
Kaplan‐Meier curve for hypothyroidism

In total, 152 (44.1%) patients developed primary HT, among whom 38 (11.0%) had clinical HT. The Kaplan‐Meier curve of HT is shown in Figure 1. The prevalence of 1‐, 2‐, and 3‐year HT was 10.7%, 25.8%, and 36.3%, respectively. Univariate analysis showed that female sex, younger age, small pretreatment thyroid volume, late N classification, high mean radiation dose, and high minimum radiation dose were risk factors for HT (Table 2).

**Table 2:** Univariate and multivariate analysis

Variable	Univariate analysis		Multivariate analysis	
	HR (95% CI)	*P*	HR (95% CI)	*P*
Age (y)		**.012**		.075
>44	0.658 (0.473, 0.916)		0.739 (0.530, 1.031)	
≤44 (reference)	1		1	
Sex		**.024**		.272
Female	1.472 (1.049, 2.065)		1.220 (0.855, 1.741)	
Male (reference)	1		1	
Pretreatment thyroid volume, cm^3^		**<.001**		**<.001**
≤16	1.995 (1.439, 2.767)		1.933 (1.388, 2.692)	
>16 (reference)	1		1	
Tumor classification^a^		.629		
T3‐4	1.085 (0.779, 1.512)			
T1‐2 (reference)	1			
Node classification^a^		**.014**		.293
N1‐3	1.867 (1.126, 3.097)		1.337 (0.778, 2.297)	
N0 (reference)	1		1	
Overall stage^a^		.325		
III‐IV	1.205 (0.831, 1.747)			
I‐II (reference)	1			
Mean radiation dose (Gy)		**<.001**	Not included	
>47	1.826 (1.322, 2.522)			
≤47 (reference)	1			
Maximum radiation dose (Gy)		.282		
>63	1.191 (0.866, 1.637)			
≤63 (reference)	1			
Minimum radiation dose (Gy)		**<.001**	Not included	
>26	2.016 (1.455, 2.793)			
≤26 (reference)	1			
Chemotherapy		.344		
Yes	1.255 (0.783, 2.010)			
No (reference)	1			
Group		**<.001**		**.001**
A (reference)	1		1	
B	2.027 (1.223, 3.358)		2.117 (1.274, 3.518)	
C	3.026 (1.796, 5.098)		2.999 (1.776, 5.064)	

*P*‐values < 0.05 are highlighted in bold.

a According to the 8th edition of the AJCC NPC staging system.

### 3.2 Dose‐volume parameters

**Figure 2:**
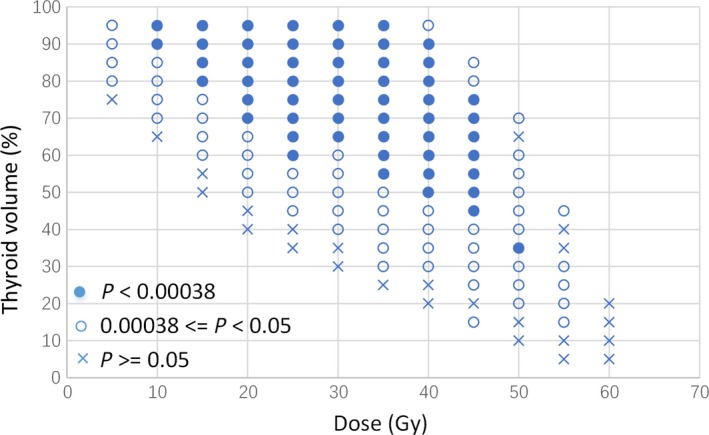
Results of univariate analysis of the association between different thyroid dose‐volume histogram parameters and hypothyroidism. An “x” represents dose‐volume combinations (*V*_Dose_ > %*V* vs *V*_Dose_ ≤ %*V*) could not discriminate hypothyroidism (*P* ≥ .05); open circles represent significant comparisons (*P* < .05); closed circles denote that comparisons remained significant under a stricter criterion of *P* < .00 038 (=0.05/133), which was required by the Bonferroni adjustment

Figure 2 shows the results of comprehensive analyses of thyroid DVH parameters. Each symbol represents a dose‐volume combination (Dose, %*V*), dividing patients into *V*_Dose_ > %*V* and *V*_Dose_ ≤ %*V* subgroups, between which HT prevalence was compared. Analysis was only carried out if a subgroup contained more than 25 patients. An ‘‘x” in Figure 2 represents a dose‐volume combination that could not discriminate HT (eg, *V*_20Gy_ > 40% vs *V*_20Gy_ ≤ 40%, *P* = .254). Open circles represent significant comparisons, with *P* < .05 (eg, *V*_20Gy_ > 50% vs *V*_20Gy_ ≤ 50%, *P* = .018). Closed circles denote that comparisons remained significant under a stricter criterion of *P* < .00 038 (=0.05/133), which was required by the Bonferroni adjustment of the *P*‐value when carrying out multiple (n = 133) analyses to define significance (eg, *V*_20Gy_ > 70% vs *V*_20Gy_ ≤ 70%, *P* < .001).

**Figure 3:**
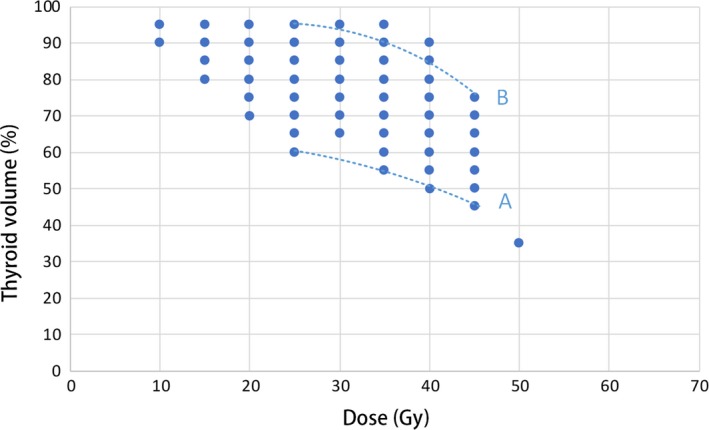
Dose‐volume histogram (DVH) constraints. Curve A: stringent thyroid DVH constraints, with *V*_25Gy_ ≤ 60%, *V*_35Gy_ ≤ 55%, and *V*_45Gy_ ≤ 45%. Curve B: inhibition curve defined by *V*_25Gy_ > 95%, *V*_35Gy_ > 90%, and *V*_45Gy_ > 75%

**Figure 4:**
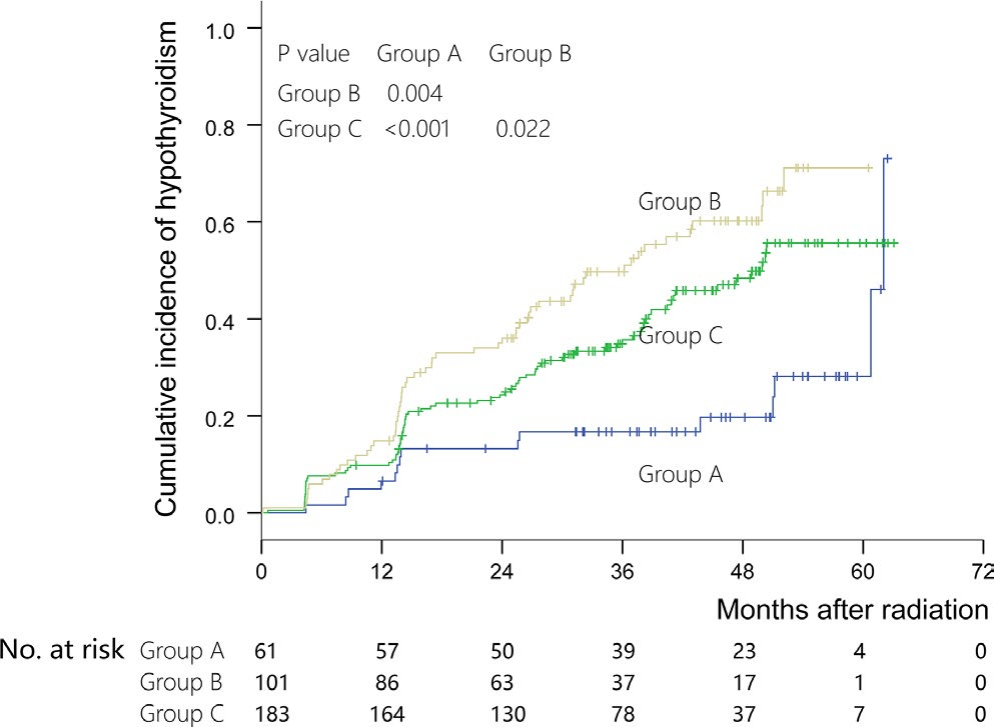
Kaplan‐Meier curve for hypothyroidism separated by group A‐C. Differences among the three groups are significant (group A vs B, *P* = .004; group B vs C, *P* = .022; group A vs C, *P* < .001)

In Figure 3, only closed circles are shown. A curve (A) was drawn to define strict thyroid DVH constraints: *V*_25Gy_ ≤ 60%, *V*_35Gy_ ≤ 55%, and *V*_45Gy_ ≤ 45%. The 2‐year prevalence of HT among patients whose DVHs met these constraints (group A) was down to 13.2% (vs overall prevalence, 25.8%, *P* < .001). Another curve (B) was drawn defined by *V*_25Gy_ > 95%, *V*_35Gy_ > 90%, and *V*_45Gy_ > 75%. The 2‐year prevalence of HT among patients whose DVHs satisfied these conditions (group B) increased to 36.0% (vs overall prevalence, 25.8%, *P* < .001). Patients not placed in group A or B were placed in group C. The 2‐year prevalence of HT of group C was 24.3%, similar to the overall prevalence. There was significant difference in HT prevalence among group A, B, or C (*P* < .05 for all) (Figure 4).

### 3.3 Multivariate analysis

Significant factors in the univariate analysis should have been included. However, there was correlation among the mean radiation dose (>47 Gy vs ≤47 Gy), minimum radiation dose (>26 Gy vs ≤26 Gy), and DVH parameters. Hence, the multivariate analysis included only sex, age (>44 years vs ≤44 years), thyroid volume (>16 cm^3^ vs ≤16 cm^3^), N classification (N0 vs N1‐3), and group A‐C defined by DVH parameters. We found that group A‐C and thyroid volume were independent factors for HT (*P* = .001 and *P* < .001, respectively) (Table 2).

## 4 DISCUSSION

This is the first study to combine several DVH parameters to identify thyroid dose‐volume thresholds for radiation‐related HT in patients treated with IMRT.

After comprehensive analyses of the association between different DVH parameters and HT prevalence, we recommended a combination of *V*_25Gy_ ≤ 60%, *V*_35Gy_ ≤ 55%, and *V*_45Gy_ ≤ 45% as the first choice of DVH constraints. Dose‐volume histograms fulfilling all three of *V*_25Gy_ > 95%, *V*_35Gy_ > 90%, and *V*_45Gy_ > 75% should be avoided if possible. These findings may help clinicians design individual treatment plans and reduce the prevalence of RT‐related HT.

Studies have reported several dose‐volume constraints, including *V*_45Gy_ ≤ 50%, *V*_50Gy_ ≤ 35%,^4^ and *V*_40Gy_ ≤ 85%.^7^ Our study found similar results. These constraints had a role in the design of treatment plans. However, only one single constraint may not be applicable for some patient subsets. For example, for NPC patients without cervical lymph‐node metastasis, several studies have reported the feasibility of lower neck‐sparing irradiation^9^, ^10^ and, in this RT method, the thyroid gland may receive less radiation, which suggests that more stringent thyroid DVH constraints are needed for such patients. For patients with a large tumor, the thyroid gland will receive a high dose unavoidably, so the DVH constraints can be relaxed appropriately but should not exceed a high level (at which the HT prevalence would be very high). Moreover, the effects of different dose levels on thyroid‐gland toxicity are not clear. Hence, using the shape of the DVH as a reference for designing treatment plans (rather than a single point on the DVH) may be more rational.

Based on the considerations made above, we undertook comprehensive analyses of different thyroid DVH parameters instead of individual dose‐volume constraints. Our results suggested that if thyroid DVHs satisfied a set of “threshold” constraints, that is, *V*_25Gy_ ≤ 60%, *V*_35Gy_ ≤ 55%, and *V*_45Gy_ ≤ 45% (together forming the “stringent line”), the prevalence of 2‐year HT was much lower than the overall prevalence; also, if thyroid DVHs met all of *V*_25Gy_ > 95%, *V*_35Gy_ > 90%, and *V*_45Gy_ > 75% (together forming the “inhibition line”), the prevalence of 2‐year HT was much higher than the overall prevalence. Moreover, for predicting HT, the stringent line was greater than any of its components (ie, the single *V*_25Gy_ ≤ 60%, *V*_35Gy_ ≤ 55%, or *V*_45Gy_ ≤ 45%); the inhibition line was greater than any of its components (ie, the single *V*_25Gy_ > 95%, *V*_35Gy_ > 90%, or *V*_45Gy_ > 75%).

According to the stringent line and inhibition line, and taking the individual illness condition and target coverage into account, clinicians can design more rational treatment plans for individuals. If possible (ie, the tumor is small and the stage is very early), the thyroid DVHs should be below the stringent line, which may help to lower the prevalence of RT‐related HT. Otherwise, putting the target coverage first, the DVH constraint can be relaxed appropriately, but thyroid DVHs should not exceed the inhibition line. Finally, in some cases (ie, the tumor is large with surrounding tissue infiltration and extensive lymph‐node metastasis in the neck), the thyroid DVH will exceed the inhibition line and the prevalence of RT‐related HT will be fairly high, which must be explained clearly by clinicians to patients. Also, regular examination of thyroid function for early detection and treatment of HT after RT is necessary.

Studies have investigated the risk factors for RT‐related HT in NPC patients, and reported similar results as our univariate analysis, including female sex,^11^ younger age,^12^ smaller pretreatment thyroid‐gland volume,^13^, ^14^ high mean radiation dose,^4^, ^15^ and high minimum radiation dose.^7^ Taking the relatively high correlation among mean radiation dose, minimum radiation dose, and DVH parameters into account, we did not include a mean radiation dose or minimum radiation dose into the multivariate analysis, and found the combined DVH constraints to be independent prognostic factors for HT. In particular, N classification (N0 vs N1‐3) was a significant factor for HT in the univariate analysis but not in the multivariate analysis. One explanation could be that the N classification (N0 vs N1‐3) is associated with the radiation dose when N0 patients usually receive lower neck‐sparing irradiation and, hence, the thyroid gland receives a lower radiation dose.

The main limitation of the present study was that the patient population was from a single center. Data from other centers are needed to provide further evidence for our conclusions. Nevertheless, we included patients receiving a uniform treatment modality, thereby avoiding the confounding effect of treatment. Moreover, the method of target delineation was uniform within these patients. Therefore, the data are comparable and our results are convincing.

In conclusion, combined thyroid DVH constraints are useful for lowering the prevalence of RT‐related HT. We recommend *V*_25Gy_ ≤ 60%, *V*_35Gy_ ≤ 55%, and *V*_45Gy_ ≤ 45% as the stringent line, and *V*_25Gy_ > 95%, *V*_35Gy_ > 90%, and *V*_45Gy_ > 75% as the inhibition line, under the precondition of not compromising the target coverage.

## Data Availability

The data that support the findings of this study are available from the corresponding author upon reasonable request.
